# Microalgae-driven microrobots: revolutionizing drug delivery and targeted therapy in biopharmaceuticals

**DOI:** 10.1007/s44307-025-00073-9

**Published:** 2025-07-01

**Authors:** Xianmin Wang, Songlin Ma, Renwu Liu, Tiexin Zhang, Xinyu Mao, Yuxue Chen, Pengcheng Wan, Zhanyou Chi, Fantao Kong

**Affiliations:** 1 MOE Key Laboratory of Bio-Intelligent Manufacturing, School of Bioengineering, Dalian University of Technology, DalianLiaoning, 116024 China; 2 Central Hospital of Dalian University of Technology, Dalian Municipal Central Hospital, DalianLiaoning, 116000 China; 3 State Key Laboratory of Fine Chemicals, School of Chemical Engineering, School of Chemistry, Dalian University of Technology, Dalian, 116024 China

**Keywords:** Microalgae, Drug delivery, Targeted therapy, Biopharmaceuticals, 3D bioprinting

## Abstract

Microalgae are a group of photosynthetic autotrophic microorganisms that are classified as Generally Recognized as safe (GRAS). They are rich in high-value bioactive compounds with broad applications in food, healthcare and pharmaceuticals. Recent research demonstrated that microalgae have significant potential as innovative biomaterials for biomedical applications. The unique phototactic movement of microalgae enables them controlled drug delivery to targeted tissues in patients. Furthermore, microalgae produce oxygen via photosynthesis when exposed to light, overcoming tumor hypoxia limitations and improving biomedical imaging in vivo. Additionally, the intrinsic biophysical properties and modifiability of microalgae can be harnessed for the development of biohybrid robots and bioprinting, expanding their clinical applications. This review highlights current engineering innovations in microalgae for medical applications, such as drug delivery, tumor hypoxia targeting, wound healing, and immunotherapy. The remarkable biocompatibility, diverse biological functionalities, and cost-effectiveness of microalgae provide a promising platform for future application of targeted drug delivery and precision medicine.

## Introduction

**Fig. 1:**
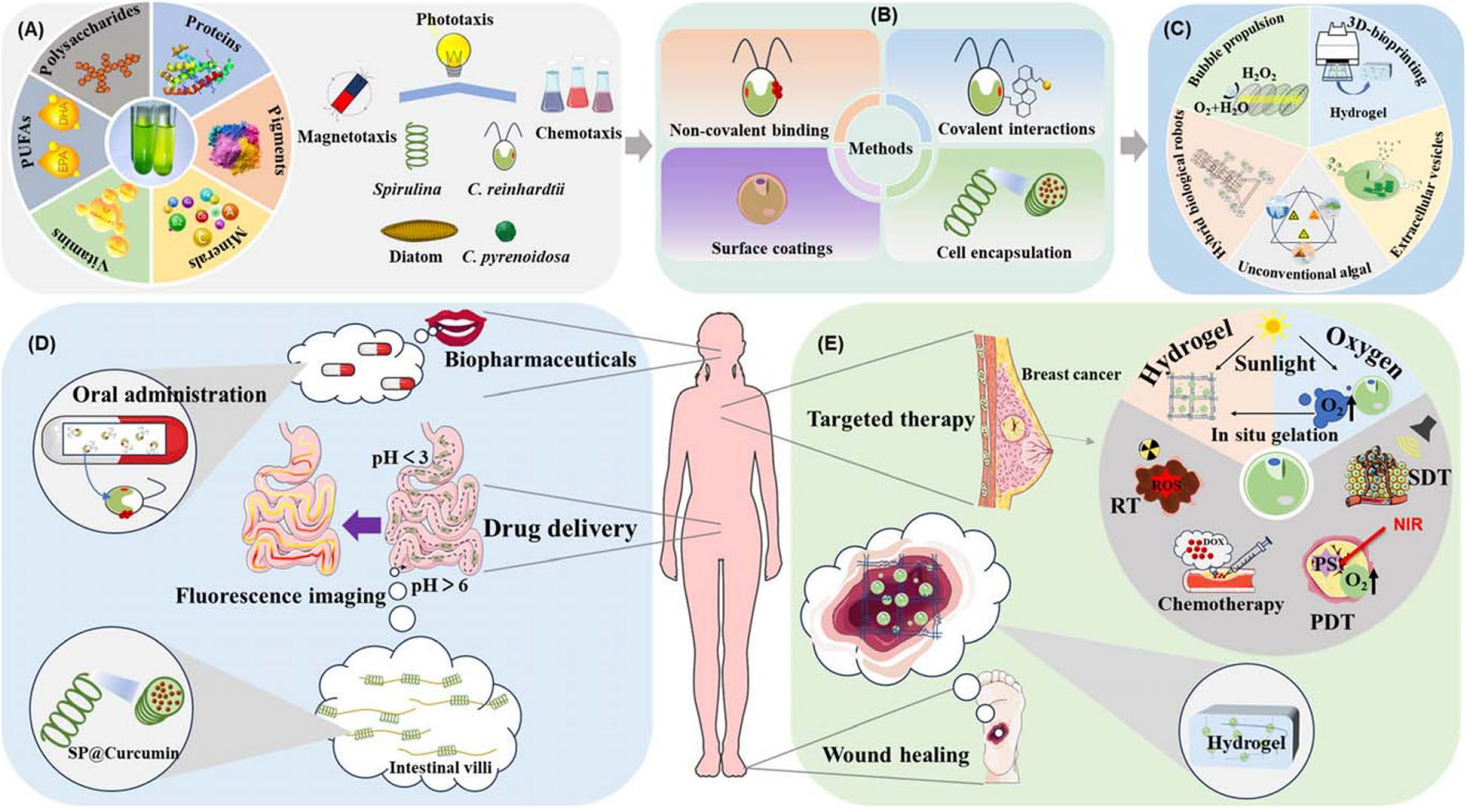
The characteristics, modifications and applications of microalgae in biopharmaceuticals. **A** The high value bioactive compounds and the directional movement of microalgae. PUFAs, polyunsaturated fatty acids. **B** The surface modifications of microalgae. **C** The unique characteristics of microalgae in specific conditions. **D** Oral administration and in vivo drug delivery by microalgae. Sp, *Spirulina*. **E** Targeted therapy and improving tumor hypoxic microenvironments by microalgae. NIR, near infrared ray; ROS, reactive oxygen species; PS, photosensitizer; DOX, doxorubicin; RT, radiotherapy; PDT, photodynamic therapy; PTT, photothermal therapy

Microalgae, the earliest photosynthetic organisms on earth, have existed for over three billion years. Due to varying climatic conditions, they exhibit significant biodiversity. More than 50, 000 microalgal species have been isolated from marine and freshwater environments (Norton et al. 1996; Uma et al. 2023). Microalgae are regarded as a valuable source of bioactive compounds with important healthcare properties, attracting interests from various industries including food, pharmaceuticals and cosmetics (Abdullah et al. 2024; Castro et al. 2023; Strizek et al. 2023; Williamson et al. 2024). Different microalgae contain varying types and amounts of bioactive substances, including lipids, vitamins, polysaccharides, phenolic compounds, polyunsaturated fatty acids (e.g., EPA and DHA), pigments (e.g., β-carotene, astaxanthin, fucoxanthin, phycocyanin, lutein, and zeaxanthin) (Occhipinti et al. 2024) (Fig. 1A). These compounds have demonstrated considerable promise in various biomedical applications, including antioxidation, anti-inflammation, anti-tumor activity, antiviral effects, and radiation protection (Bouyahya et al. 2024; Hernandez-Urcera et al. 2024; Huang et al. 2024; Lee et al. 2016; Zhao et al. 2024).

To adapt to the complex changes in natural environments, microalgae have developed sensory capabilities and directional movement (Chu et al. 2022). The sizes of microalgae range from a few micrometers to several hundred micrometers, and their motility is influenced by both species and cellular structure (Zhang et al. 2024c) (Fig. 1A). For example, the flagella of *Chlamydomonas reinhardtii* (*C. reinhardtii*) can beat at frequencies of 60–70 Hz, reaching speeds of several hundred micrometers per second (Geyer et al. 2013). The directional movement of microalgae is mainly classified into phototaxis, magnetotaxis and chemotaxis (Gotovtsev 2023). These distinctive locomotion behaviors hold great promise for the studies of biomedicine and microenvironmental drug transportation (Akolpoglu et al. 2020; Ozasa et al. 2017; Urso et al. 2023).

Microalgae are capable of detecting light stimuli via eyespots and can regulate their movement through flagellar beating, resulting in either positive or negative phototactic responses (Song et al. 2023; Ueki et al. 2016; Xie et al. 2016; Xin et al. 2020). Based on this principle, Weibel et al. (2005) created light-driven biological micromotors using microalgae. These microalgae-based micromotors can be employed for the targeted delivery of micron-sized cargo using light. Furthermore, Xie et al. (2021) developed automated algal robots that are capable of precise adhesion and movement of algal cells through light control. The wild-type (WT) algal strains mainly respond to ultraviolet and visible light. However, the potential risks of light-induced damage and the limitations of penetration associated with these wavelengths limited the application of these WT strains. Near-infrared light therapy has emerged as the preferred method for phototherapy. The development of red-shifted light-sensitive proteins or nanomaterials for light converting will be promising for advancing light-targeted therapies using microalgae.

Magnetism exhibits stronger penetration and higher safety compared to light, making it more widely applicable in the biomedical field (Zhang et al. 2024d). However, WT algal strains do not exhibit magnetotaxis. To date, magnetic-driven micromachines utilized in medicine have primarily involved magnetotactic bacteria (MTB) (Bazylinski et al. 2013; Ren et al. 2023; Salam et al. 2023). However, the high cultivation costs of MTB, along with unverified biocompatibility and diminished effectiveness as transport proteins, pose significant challenges (Bazylinski et al. 2013). In contrast, microalgae can be engineered to attach magnets, realizing magnetotactic movement when subjected to an external magnetic field. For instance, magnetite nanoparticles were assembled on the surface of microalga *Spirulina* to form helical micro/nano swimming robots, which can be used as advanced medical tools (Li et al. 2024b; Xie et al. 2020; Zhuge et al. 2022). Additionally, Santomauro et al. (2018) reported that incorporated terbium into microalgae is sufficient for achieving internal magnetization, enabling directed movement.

Chemotaxis is the directional movement of organisms in response to gradients of chemical stimuli, including both nutrients and toxic compounds (Young and Mitchell 1973). Previous studies predominantly focus on bacterial chemotaxis, however studies on microalgal chemotaxis are still limited. It was reported that *C. reinhardtii* responds chemotactically to HCO3^−^ (Choi et al. 2016). The chemotactic movements that microalgae evolved in environments can offer valuable insights for designing biomedical micromachines. Microalgae also display other directional behaviors that are worthy of further exploration, such as aerotaxis and gravitaxis (Gotovtsev 2023; Haeder and Hemmersbach 2022). Overall, the targeted propulsion of such micromachines, which can be controlled by light, magnetic fields, ultrasound, heat, or other external stimuli that affect microalgal behavior (Ishikawa et al., 2023; Maria-Hormigos et al. 2022). These studies could significantly expand the applications of microalgae in biomedicine.

Advances in nanotechnology and materials science have accelerated progress in micro-robotics, unlocking novel possibilities for biomedical applications. While synthetic biohybrid robots often use metal or polymer frameworks, their practical use faces constraints such as limited biocompatibility, difficulty targeting specific organs/tissues, unstable performance in biological fluids, and costly manufacturing. These challenges have driven increased focus on natural biomaterials as foundational platforms for next-generation micro-robotic systems (Murali et al. 2024; Oda et al. 2024). During the past years, a variety of microorganisms have been employed, including motor proteins, muscle tissues, sperm cells and bacteria (Xu et al. 2018). Recently, microalgae have attracted great attentions as potential natural biomaterials.

Microalgae have a variety of shapes, such as spherical, spiral, oval, and other irregular forms, which facilitate the development of micro/nano robots for diverse applications (Fig. 1AandTable 1). For example, the uniformly spherical *Chlorella pyrenoidosa*, with a diameter of 3–5 μm, is even smaller than human red blood cells (Wu et al. 2023). *Chlorella*'s remarkable microscale size, combined with its uniform structure and excellent biocompatibility, makes it an ideal biological template to construct hybrid micromachines for targeted cancer therapy (De Gong et al. 2022; Gao et al. 2023; Lee et al. 2019; Liang et al. 2024a; Qiao et al. 2020; Zhong et al. 2021a). *Spirulina*, named for its distinctive 3D spiral shape, features an exceptionally high specific surface area that not only facilitates magnet attachment and drug loading (An et al. 2024; Hua et al. 2024; Li et al. 2024b; Wang et al. 2019; Zhong et al. 2020), but also enhances capture by intestinal villi for treating gastrointestinal diseases (Liang et al. 2024b; Zhang et al. 2023, 2022a; Zhong et al. 2021b). The porous silica microcapsules produced by diatoms can serve as natural drug carriers for delivery applications (Aw et al. 2012; Maher et al. 2016; Sasirekha et al. 2019). Additionally, the natural bio-silica found in diatom cell walls, serves as an excellent material for bone tissue repair (Cicco et al. 2019; Dalgic et al. 2019; Delalat et al. 2015; Le et al. 2016; Maher et al. 2016; Sasirekha et al. 2019; Sun et al. 2024a). *C. reinhardtii*, a widely utilized model organism, is also employed in the development of hybrid biological robots (Akolpoglu et al. 2020; Santomauro et al. 2018; Shchelik et al. 2021; Zhang et al. 2022c). For instance, the cell wall-less strains of *C. reinhardtii* can be used in biomedicine for screening anti-cancer drugs, thereby reducing the complex and costly culture requirements associated with mammalian cells (Maucourt et al. 2002). Furthermore, the significant accumulation of carboxyl, hydroxyl, and amine functional groups on the surfaces of microalgal cell walls results in their negatively charged surface (Li et al. 2022). This property enables them to function as carriers for drugs, such as positively charged doxorubicin for medical delivery applications (Akolpoglu et al. 2020; Boalwe et al. 2015).

**Table 1:** Microalgae-based micro/nano robots tailored for medical applications in recent years

Microalgal species	Engineered approaches	Delivered cargos	Targeted approaches	Medical applications	References
*C. pyrenoidosa*	Electrostatic adsorption	Doxorubicin	In situ injection	Treatment of osteosarcoma	(Liang et al. 2024a)
	Electroless deposition	Doxorubicin	Magnetic propulsion	Enhanced targeted drug delivery	(De Gong et al. 2022)
	Calcium phosphate coating	—	Active dispersion	Addressing radioresistance in hypoxic tumors	(Zhong et al. 2021a)
	Cell membrane wrapping Cell membrane wrapping	—	Active dispersion	Addressing radioresistance in hypoxic tumors	(Qiao et al. 2020)
		Chloroquine diphosphate and Hematoporphyrin dihydrochloride	Immune targeting	Enhanced anti-tumor immunotherapy	(Gao et al. 2023)
	Hydrogel	Doxorubicin	In situ injection	Treatment of hypoxic breast cancer	(Lee et al. 2019)
*Spirulina*	Electrostatic adsorption Electrostatic adsorption Electrostatic adsorption	Doxorubicin	Passively target by pulmonary capillaries	Treatment of breast cancer lung metastases	(Zhong et al. 2020)
		Doxorubicin	In situ injection	Treatment of osteosarcoma	(An et al. 2024)
		Astaxanthin	Passive capture by intestinal villi	Against radiation-Induced Injury	(Zhang et al. 2023)
	Cell encapsulation Cell encapsulation Cell encapsulation	Curcumin	Passive capture by intestinal villi	Oral drug delivery therapy for intestinal diseases	(Zhong et al. 2021b)
		Amifostine	Passive capture by intestinal villi	Intestinal radiotherapy protection	(Zhang et al. 2022a)
		Metformin	In situ injection	Osteoarthritis treatment	(Liang et al. 2024b)
	Electroless deposition	Doxorubicin	Magnetic propulsion	Targeted delivery and synergistic chemo-photothermal therapy	(Wang et al. 2019)
	Hydrogel	Resveratrol	Floating in gastric fluid	Alcohol-induced diseases treatment	(Hua et al. 2024)
*T. pseudonana*	Genetic engineering	Camptothecin and 7-ethyl-10-hydroxy-CPT	Immune targeting	Targeted delivery of poorly water-soluble drugs	(Delalat et al. 2015)
*T. weissflogii*	Cellular infiltration	Sodium alendronate	—	Local activation of bone cells	(Cicco et al. 2019)
*A. subtropica*	Chemical modification	Doxorubicin	—	Delivery of anti-cancer drugs	(Sasirekha et al. 2019)
*C. reinhardtii*	Electrostatic adsorption	Doxorubicin	Light	Transport of medical cargo	(Akolpoglu et al. 2020)
	Click chemistry	Ciprofloxacin	Passively target by pulmonary capillaries	Treatment of acute bacterial pneumonia	(Zhang et al. 2022c)
	Click chemistry	Vancomycin and Ciprofloxacin	Light	Against bacterial infections	(Shchelik et al. 2021)
	Cellular infiltration	Medicines	Magnetic propulsion	Transport of medical goods, endoluminescent imaging	(Santomauro et al. 2018)

‘—’ indicates the either delivered cargos or targeted approaches were not used

Microalgae can be easily functionalized using various methods, including non-covalent binding, covalent interactions, and additional techniques such as surface coatings, cell permeation, and encapsulation (Akolpoglu et al. 2020; Qiao et al. 2020; Zhang et al. 2022a, 2022c) (Fig. 1B). This functionalization significantly enhances the potential medical applications of microalgae as micromachines. Furthermore, advancements in state-of-the-art genetic engineering tools, such as CRISPR/Cas9, are now available for microalgae (Kong et al. 2024, 2019), which will be beneficial for medical applications of microalgae.

## The unique characteristics of microalgae in specific conditions

### In vivo fluorescence imaging O_2_ production and bubble propulsion

Microalgae contain photosynthetic pigments, such as chlorophyll and carotenoids (Aizpuru et al., 2024). These pigments enable microalgae to exhibit spontaneous fluorescence and produce O_2_ through photosynthesis under light illumination. For instance, the chlorophyll in microalgae emits red fluorescence at specific excitation wavelengths, similar to the artificial fluorescent dye (Zhong et al. 2020). This property makes microalgae as a promising candidate for in vivo fluorescence imaging (Zhong et al. 2020). Additionally, the ability of microalgae to produce O_2_ through photosynthesis exhibited extensive applications in hypoxic environments, including tumor microenvironments, and wound healing (He et al. 2023; Ma et al. 2024; Zhong et al. 2021a). In addition to photosynthetic O_2_ production, microalgae have the ability to catalyze the decomposition of H_2_O_2_ used as fuel to prepare self-propelled micromotors (Panda et al. 2018) (Fig. 1C). The cell walls of diatoms are mainly made up of SiO_2_, Fe_2_O_3_, and Al_2_O_3_, enable the conversion of Fe_2_O_3_ to Fe_3_O_4_, which can catalyze the decomposition of H_2_O_2_ fuel to generate bubble propulsion (Panda et al. 2018). The movement of diatom motors can be tailored by cleaving EDTA molecules to obstruct catalytic sites on the motors, thereby broadening the potential applications of self-propelled micro-motors (Panda et al. 2018).

### Three D bioprinting using microalgae

3D bioprinting has emerged as a disruptive technology in the field of biomedicine, with broad applications in tissue engineering and regeneration, disease modeling, personalized medicine, drug discovery, and drug delivery (Nguyen et al. 2018). Recently, there has been growing interests in the 3D bioprinting using microalgae (Fig. 1C). These interests are partially due to their rich pharmacologically active compounds, rapid growth rates, temperature resilience, adaptability to harsh environments, and the capability for localized O_2_ release (Krujatz et al. 2015; Morita et al. 2000; Sun et al. 2024b). 3D bioprinting using microalgae to fabricate three-dimensional scaffolds, tissues, and organ structures is more natural and cell-friendly. The resulting hydrogels are capable of absorbing O_2_, H_2_O, and nutrients while providing excellent printability, biocompatibility, and biodegradability (Malik et al. 2020; Raees et al. 2023).

Recently, 3D bioprinting immobilization is increasingly being applied in the biomedical treatment, such as tumor therapy, wound healing and tissue repair (Wang et al. 2023). However, engineered living materials (ELM) created by 3D bioprinting fixation do not simply increase the total volume of the ELM for higher functionality or productivity. To predict and enhance the functionality of these photosynthetic ELM, Oh et al. (2023) explored the growth, distribution and photosynthesis of *C. reinhardtii* in 3D hydrogels, and found that the three-dimensional map of algal growth within the ELM. The availability of carbon sources, and the light penetration are limiting factors for algal cell growth (Oh et al. 2023). Molding the ELMs into a thin leaf-like structure maximizes the surface-to-volume ratio and paves the way for the novel photosynthetic material development using microalgae (Oh et al. 2023). Nonetheless, most of these bioprinted biomaterials still lack sufficient mechanical strength. Recently, Balasubramanian et al. (2021) found that bacterial cellulose, characterized by its nanofiber structure and absorption capacity, enhances nutrient diffusion to microalgal cells, thereby promoting their growth. This study integrated the photosynthetic capabilities of microalgae with the physical and mechanical properties of bacterial cellulose, providing alternative opportunities for regenerative photosynthetic biomaterials. To investigate the impact of bioprinting on microalgae, Cui et al. (2024) tested three microalgal strains (*C. sorokiniana*, *C. reinhardtii*, and *Synechococcus* sp.) using bioprinting and jetting techniques. They found that bioprinting do not adversely affect the cell morphology, viability, or photosynthetic efficiency of microalgae (Cui et al. 2024). Overall, the biomaterials (beads and fibers/scaffolds) containing microalgae show promise for wound healing therapies.

### Algal extracellular vesicles for biomedicals

Extracellular vesicles (EVs) encompass a diverse range of membrane structures released by cells, including exosomes, microvesicles, microparticles, and apoptotic bodies. They are regarded as promising biological nanocarriers for delivering both natural and exogenous molecular cargos (Adamo et al. 2024; Thery et al. 2018). Recently, there has been growing interests in microalgae-derived EVs (Fig. 1C). Adamo et al. (2021) isolated a type of"nanoalga"from cultures of various microalgal strains, referring to it as microalgal EVs. Furthermore, they found that these algal EVs could be effectively and safely absorbed by mammalian cells, confirming their potential for cross-talk communication (Adamo et al. 2021). Recently, Adamo et al. (2024) discovered that EVs derived from the microalga *Tetraselmis chuii* demonstrated excellent biocompatibility and unique osteotropic properties, along with antioxidant and anti-inflammatory characteristics (Adamo et al. 2024). Compared to mammalian cell culture systems, microalgae offer a promising solution to the current challenges of exosome-based drug carriers due to their rapid growth rate, simple culture conditions, and scalability in EVs production, indicating significant potential for future biomedical applications (Adamo et al. 2025). Intriguingly, microalgal EVs can encapsulate and deliver metabolites, such as proteins and nucleic acids, preserving their original pharmacological effects (Picciotto et al. 2021; Hu et al. 2024). For instance, EVs isolated from *Haematococcus pluvialis* (HpEVs) can efficiently deliver lipid-soluble compounds like astaxanthin, enhancing their bioavailability and tissue-specific therapeutic effects (Hu et al. 2024). Similarly, EVs derived from *Spirulina platensis* have been shown to mitigate osteoarthritis (OA)-associated inflammation by scavenging reactive oxygen species (ROS) and downregulating pro-inflammatory cytokines (e.g., IL-6), highlighting their potential as a novel OA therapy (Liang et al. 2025). To further validate microalgae as promising EV producers, Picciotto et al. (2021) analyzed nanosized extracellular nano-objects generated by various microalgal species. They identified seven promising strains capable of producing EVs from distinct lineages, demonstrating microalgae as a novel and sustainable source of EVs (Picciotto et al. 2021). It was reported that microalgal EVs remain structurally and functionally stable under extreme conditions (e.g., high light and salt stress). For instance, *Haematococcus pluvialis* EVs produced under high light stress contain abundant antioxidant proteins and metabolic regulators, retaining their activity even during prolonged in vitro storage (Hu et al. 2024). These findings demonstrate that microalgal EVs possess unique pharmacological properties and efficient cargo delivery capabilities, highlighting their significant potential for clinical applications.

## Unlocking the medical potential of unconventional algal strains

The unconventional algal strains, such as extremophilic microalgae, have also shown significant potential for biomedical applications (Gao et al. 2024; Occhipinti et al. 2024; Varshney et al. 2015) (Fig. 1C). Extremophilic algae thrive in harsh conditions, such as extreme pH levels, high CO_2_ concentrations, extreme temperatures, high salinities, and radiation. These algae have evolved unique motility behaviors and metabolic pathways (Rojas-Villalta et al. 2024). Recently, Zeng et al. (2023) identified an Antarctic ice microalga (AIM) that thrives in fixed and mobile ice regions of the Southern Ocean. They combined the polypeptides from AIM (AIMP) with selenium nanoparticles (SeNPs) as modifiers, resulting in AIMP-SeNPs (Zeng et al. 2023). AIMP-SeNPs act as effective antioxidants and have the potential to replace traditional antibiotics as highly effective antibacterial agents in healthcare (Abiusi et al. 2022). It was reported that acid-tolerant and acidophilic microalgae exhibit reduced risks of microbial contamination and biomass productivity comparable to neutrophilic *Chlorella*. This lays a solid foundation for the application of microalgae in biomedicine. Meanwhile, Zhang et al. (2022b) found that algal biohybrid robots based on the acidophilic alga *C. pitschmannii* can be functionalized by various carriers and applied in harsh environments such as acidic biofluids and gastric juices, which offer considerable promise for gastrointestinal drug delivery. Although some bioactive substances produced by microalgae carry significant risks due to their high toxicity, they may provide therapeutic benefits. For instance, algal toxins produced by microalgae exhibit notable pharmacological activities, such as anti-tumor and anti-inflammatory effects, making them potential candidates for treating various diseases (Gao et al. 2024). Moreover, *Microcystis aeruginosa* (MA) has been recognized as a toxic microalga due to its production of microcystin toxins, which pose threats to animal and human health. However, Meng et al. (2024) recently revealed that microcystin from MA possesses potential immunomodulatory properties. When the engineered MA@PDA-F127 was injected into tumors, it rapidly transformed into gel form and localized within the tumors, avoiding toxic effects on surrounding tissues (Meng et al. 2024). This hydrogel offers effective anti-tumor treatment by enhancing tumor immunogenicity and activating systemic immune responses, paving the way for the development of tumor therapies based on toxic microalgae.

## Applications of microalgae in biomedicine

### Antioxidants and nutritional benefits

Microalgae are classified as generally recognized as safe (GRAS) by the US Food & Drug Administration (FDA) (Castillo et al. 2023). *Spirulina* (Cyanophyta) and *Chlorella* (Chlorophyta) have been cultivated on a large scale and widely used in functional foods and dietary supplements (Gouda et al. 2022). *Spirulina* possess high protein content and exceptional nutritional value. It not only provides essential fatty acids that the human body cannot synthesize but also serves as a functional food that nourishes beneficial gut microbiota, including *Lactobacillus* and *Bifidobacterium* (Sathasivam et al. 2019). *H. pluvialis* is the major natural producers of astaxanthin, a fat-soluble carotenoid known for its high antioxidant properties and various health benefits (Liu et al. 2014; Tambat et al. 2023). The antioxidant capacity of astaxanthin is 10-times greater than that of β-carotene, lutein, and zeaxanthin. Niu et al. (2020) investigated the effects of astaxanthin derived from *H. pluvialis* in pregnant mice. They found that astaxanthin reduced low-density lipoproteins (LDL) and total cholesterol levels with no adverse effects (Niu et al. 2020). The green microalga *D. salina* contains carotenoids (9-cis-β-carotene) known to prevent intracellular oxidative damage and can be consumed as a dietary supplement for human health or as an ingredient in natural foods and beverages (Yaakob et al. 2014). The paramylon (β−1,3-D-glucan) found in *Euglena* has immune-stimulating effects involving cytokines that can alleviate allergic diseases. Hydrothermal extracts of *Euglena* have been shown to reduce viral intensity of various influenza viruses in vitro (Isegawa 2023). A study on the impact of *Euglena* intake on gut microbiota and bowel movements indicated that *Euglena* increased the abundance of the genus *Faecalibacterium* (Nakashima et al. 2021), showcasing its potential as a novel prebiotic. Similarly, a study by Kawano et al. demonstrated that supplementation with *Euglena gracilis*-derived paramylon significantly reduced fatigue in healthy adults (Kawano et al. 2020). Recently, Fields et al. (2020) examined the effects of *Rhodomonas* on gastrointestinal health. They found that feeding *Rhodomona*s to mice with acute colitis alleviated weight loss, positively influencing gastrointestinal symptoms in humans (Fields et al. 2020).

### Targeted drug delivery

In recent years, the global incidence of cancer has steadily risen, posing a significant threat to human health (Andrade et al. 2018). Chemotherapy, a widely used treatment method, faces considerable limitations in its clinical effectiveness. This is partially due to the rapid metabolism of antitumor drugs and their poor penetration into deep tumor tissues (Shahriar et al. 2022). To overcome these challenges, researchers are exploring microalgae as natural carriers, which offer excellent biocompatibility, high propulsion capabilities, and cargo-carrying potential. Notably, microalgae are naturally occurring organisms with cell walls composed of materials such as cellulose and polysaccharides that are inherently biocompatible, minimizing immunogenic responses. Moreover, these cell walls can be naturally degraded in vivo by enzymes or intestinal microbes, avoiding the drawbacks of synthetic materials (e.g., certain polymers) that may accumulate or trigger inflammation, thereby reducing the risk of long-term toxicity (Zhang et al. 2024e). Compared to bacteria-driven micromotors, microalgae-driven micromachines have diameters over five times larger, enabling faster speeds and more precise directional control for drug delivery in liquid environments (Oda et al. 2024). Additionally, the unique cellular structure of microalgae-such as their cell walls and vesicles-can accommodate both hydrophilic and hydrophobic drugs, making them suitable for loading a wide variety of drug types (Zhang et al. 2024c). Micro-robots powered by microalgae can significantly improve targeted drug delivery. This review highlights *C. reinhardtii* and *Spirulina* as examples, discussing relevant modification techniques for microalgae and the applications of micro-robots developed from these modifications in drug delivery across various disease models.

#### In vivo drug delivery using C. reinhardtii

*C. reinhardtii*, a biflagellate and phototropic green microalga, features natural autofluorescence that enables label-free fluorescence imaging in biological systems and allows for surface modifications to carry cargo on its cell wall (Wang et al. 2018) (Fig. 1D). Zhang et al. (2022c) modified *C. reinhardtii* with nanoparticles to create micro-robots for in vivo antibiotic delivery targeting acute bacterial pneumonia. They modified the algal surface using azide-N-hydroxysuccinimide ester, and neutrophil membrane-coated poly (lactic-co-glycolic acid) was concurrently prepared as an effective antibiotic carrier (Zhang et al. 2022c). A click chemistry reaction based on azido-dibenzocyclooctyne subsequently linked the antibiotic-loaded polymer nanoparticles to the microalgae (Zhang et al. 2022c). These results indicated that micro-robots could rapidly and uniformly distribute in simulated lung fluid (> 110 μm/s) and exhibited low clearance rates by alveolar macrophages, allowing for extended retention. In mouse models of acute *Pseudomonas aeruginosa pneumonia*, significant therapeutic effects were observed after treatment, indicating favorable biosafety of these micro-robots (Zhang et al. 2022c).

Qiao et al. (2020) employed natural, untreated red blood cell membranes to coat microalgae. This method effectively reduced macrophage uptake and immune clearance while preserving the microalgae's efficient photosynthetic O_2_ production (Qiao et al. 2020). Furthermore, Gao et al. (2023) modified this approach by coating engineered microalgae with macrophage membranes, thereby improving biocompatibility and utilizing the membranes'inflammatory homing capabilities for targeted delivery to tumors. These results suggest the potential for using other natural human cell membranes, such as those from cancer cells or immune cells to wrap microalgae (Jin et al., 2020; Zeng et al. 2024). It may enhance the biocompatibility and targeting abilities of micro-robots in future applications.

The functionalization of microalgae through covalent bonding is also a promising approach for developing biohybrid robots that can achieve long-term stability and resilience in harsh environments. Zhang et al. (2021) functionalized *Rhizoclonium* with angiotensin-converting enzyme 2 (ACE2) receptors targeting the SARS-CoV-2 spike protein through click chemistry. The ACE2 microalgae robots achieved rapid (> 100 μm/s) and sustained (> 24 h) self-propulsion (Zhang et al. 2021), making them promising candidates for virus removal and offering substantial potential for drug delivery.

The strategy of using covalent bonding to attach drugs has resulted in lower-than-anticipated manufacturing efficiency and heightened technical complexity, making it impractical for large-scale production. To address this challenge, Akolpoglu et al. (2020) developed a technique that involves applying a chitosan coating to the cell walls of *Rhizoclonium*, thereby forming a thin protective layer (Akolpoglu et al. 2020). They then used a photolabile linker to couple embedded iron oxide nanoparticles with the chemotherapy drug doxorubicin, resulting in biohybrid robots capable of on-demand drug release using ultraviolet light for breast cancer treatment (Akolpoglu et al. 2020). This approach leveraged electrostatic interactions to simplify the manufacturing process, achieving an impressive 90% efficiency in producing biohybrid micro-machines (Akolpoglu et al. 2020). Notably, these engineered microalgae still maintained their motility and phototactic behavior.

#### Treatment of gastrointestinal diseases with spirulina platensis

*Spirulina platensis* (*Spirulina*) is a natural microalga that thrives in alkaline environments and has a three-dimensional spiral shape. It is well-known as its rich active components and is now used as supplements for food, pharmaceuticals, and biomedicine (Luo et al. 2024a; Shah et al. 2024; Zhang et al. 2023). Recently, *Spirulina* was found to be negatively charged with high specific surface area, which effectively accommodates oppositely charged drug molecules. Its unique structure also show potential for navigating complex biological environments, making it particularly promising for the treatment of gastrointestinal diseases (Fig. 1D).

Currently, oral administration is the preferred method for treating gastrointestinal diseases. However, the acidic environment of the stomach often leads to rapid degradation of drug molecules during direct oral delivery (Karamanidou et al. 2016). This hinders their retention in the intestine and significantly reduces therapeutic efficacy. To improve oral delivery, Zhong et al. (2021b) developed a microalgae-based drug delivery system utilizing *Spirulina* for various intestinal diseases. This system involves loading curcumin onto *Spirulina*, followed by centrifugation to prepare the formulation (Zhong et al. 2021b). The chlorophyll in *Spirulina* also enables non-invasive fluorescence imaging in vivo, making this method suitable for large-scale production. In terms of therapeutic efficacy, *Spirulina* loaded with curcumin (SP@Curcumin) maintains its integrity in the stomach. SP@Curcumin is subsequently captured by intestinal villi, where it gradually degrades and releases curcumin for targeted and sustained drug delivery (Zhong et al. 2021b). SP@Curcumin not only shows synergistic effects when combined with chemotherapy and radiotherapy for colon cancer, but also exhibits anti-inflammatory properties. Similarly, Zhang et al. (2023) designed an oral microalgae nano-integration system for intestinal and systemic radioprotection. They loaded astaxanthin (ASX) into poly (lactic-co-glycolic acid) (PLGA) to create ASX@PLGA particles, which were then coated with chitosan to form ASX nano-particles (Zhang et al. 2023). The electrostatic attraction between the positively charged ASX nano-particles and negatively charged *Spirulina* enabled the formation of a drug delivery system (SP@ASXnano). This system effectively combines *Spirulina*'s ability to prolong intestinal retention and provide controlled drug release with ASX nano's properties. This strategy enhanced drug solubility, gastric stability, cellular uptake, and intestinal absorption (Zhang et al. 2023). These studies indicated that microalgae act as a versatile platform for delivering various medications to treat different diseases.

In addition to direct gastrointestinal disorders, a range of alcohol-related conditions, such as gastric bleeding and gastrointestinal inflammation, have gained significant attention. Recently, Hua et al. (2024) created an innovative natural floating drug delivery system based on microalgae, aimed at treating alcoholic diseases. They combined needle-like rod-shaped resveratrol (RSV), known for its anti-inflammatory and antioxidant properties, with SP to create a stable structure called SP-RSV (Hua et al. 2024). Furthermore, they incorporated acid-sensitive pectin-bismuth to develop a mesh-like floating biological system known as SP-RSV-Bi. Pectin-bismuth will form a gel only when it contacts simulated gastric fluid (SGF), which alleviates swallowing difficulties for patients and significantly extending the floating duration of SP-RSV in SGF (Hua et al. 2024). This system also rapidly releases RSV upon exposure to alcohol, demonstrating effective therapeutic outcomes. Although *Spirulina*-based drug delivery systems have been successfully developed for the treatment of gastrointestinal diseases, the potential of *Spirulina*'s application may go beyond that. For example, recently, Liang et al. (2024b) created a micro-bioreactor (SP@Met) for the treatment of osteoarthritis by utilizing *Spirulina* as a drug carrier for effective loading of metformin. In addition, whether microalgae with enhanced floating abilities to deliver a variety of drugs that can be released or absorbed in the stomach for the treatment of various gastric diseases should be explored in the future.

#### Other microalgae for drug delivery

It was reported that diatoms, marine unicellular microalgae, also are promising for drug delivery. Their outer shell, diatom biosilica (DB), is a natural inorganic material with a hierarchical micro-nano porous structure. This unique multi-scale porosity enables DB to serve as an excellent drug carrier. (Sun et al. 2024a). Cicco et al. (2019) demonstrated successful incorporation of alendronate into the DB of *Thalassiosira weissflogii* through in vivo feeding, establishing this approach as viable for drug loading with potential applications in bone tissue regeneration (Cicco et al. 2019). Diatoms offer a novel approach to improve the bioavailability of water-soluble drugs. For instance, Gnanamoorthy et al. (2014) encapsulated the hydrophilic antibiotic streptomycin within *Coscinodiscus concinnus* biosilica, which protected the drug from degradation while enabling sustained release for prolonged antimicrobial activity. (Gnanamoorthy et al. 2014).

Beyond functioning as simple drug carriers, microalgae can actively migrate to target sites and secrete bioactive compounds to exert therapeutic effects. For instance, several studies demonstrate that orally administered microalgae can both modulate gut microbiota and release endogenous antioxidant and anti-inflammatory components, making them effective for treating gastrointestinal disorders (Zhong et al. 2024; Novichkova et al. 2023; Castro et al. 2020; Yang et al. 2025).

### Improving the tumor hypoxic microenvironment

Although chemotherapy is a commonly used for cancer treatment, this method inevitably damages normal cells. To overcome these challenges, the strategies such as radiotherapy (RT), photodynamic therapy (PDT), photothermal therapy (PTT), and biologically targeted drug delivery combined with RT have been introduced to combat malignant tumors (Barker et al. 2015; Liu et al. 2024). However, the rapid growth of tumors, particularly in areas such as blood vessels, inevitably leads to localized hypoxia, creating a hypoxic microenvironment that restricts the effectiveness of these treatments. The main reason for this limitation is the requirement for oxygen in the cell-killing process (Maas et al. 2012). Therefore, reoxygenation of the tumor hypoxic microenvironment will be an effective method to overcome these difficulties. Various nanocarriers that supply or generate oxygen at cancer sites, such as hemoglobin-based oxygen carriers, perfluorocarbon-based O_2_ carriers, and catalase-mediated oxygen generation were developed (Li et al. 2024a; Luo et al. 2024b). Although those nanotherapy shows great potential in alleviating tumor hypoxia, issues related to biocompatibility, safety, and targeting within complex tumor microenvironments remain inherent limitations. Microalgae represent a novel biomaterial that uniquely combines photosynthetic oxygen production with localized CO_2_ absorption. Unlike conventional oxygen-releasing materials, they can simultaneously alleviate tumor hypoxia while consuming surrounding carbon dioxide, thereby enhancing the effectiveness of radiotherapy RT, PDT and PTT (Wang et al. 2024b, 2021; Zhang et al. 2024b; Zhong et al. 2021a) (Fig. 1E). Among these microalgae-based therapeutic methods, the most commonly used microalga is *Chlorella*, so this section will primarily focus on *Chlorella*, with other types of microalgae discussed as supplementary.

#### Application of chlorella in tumor hypoxic microenvironments

*Chlorella*, a spherical green microalga, produces a significant amount of reactive oxygen species (ROS) when exposed to 650 nm laser irradiation, making it an attracting material for treating hypoxic tumors. Qiao et al. (2020) engineered *Chlorella* with natural red blood cell membranes to create a novel oxygen-generating system. This microalgae-mediated in situ oxygen production system significantly improved the hypoxic conditions within tumors and resulted in notable enhancements in radiotherapy efficacy (Qiao et al. 2020). Additionally, *Chlorella* can be surface-coated with various substances. For example, *Chlorella* was coated with calcium phosphate (CaP) to allow the algae to reach tumor sites while preserving its photosynthetic activity under harsh environments (Zhong et al. 2021a). This biomimetic system (CV@CaP) not only continuously generates O_2_ in situ within the tumor, but also demonstrates excellent fluorescence and photoacoustic imaging properties, thereby improving radiotherapy outcomes and enabling self-monitoring (Zhong et al. 2021a). Similarly, *Chlorella* was combined with alginate using calcium cross-linking to create an autotrophic light-triggered green oxygen supply engine that continuously generates oxygen around tumors, facilitating repeated and efficient PDT (Zhou et al. 2019). Furthermore, ultrasound-triggered sonodynamic therapy (SDT) is another non-invasive cancer treatment approach. In a recent study, *Chlorella* (Chl) was encapsulated within macrophage membranes to form MChl and subsequently coupled MChl with drug-loaded nanoliposomes (NP) (Gao et al. 2023). This coupling was achieved through interactions between β-CD-modified MChl and ADA-modified liposomes, resulting in MChl-NP targeting melanoma and ultimately enhancing SDT efficacy by alleviating hypoxia and inhibiting autophagy (Gao et al. 2023). Additionally, combining the photosynthetic O_2_-producing properties of *Chlorella* with the in situ immobilization properties of hydrogels to enhance hypoxia-limited tumor therapy would be another extremely innovative strategy (Lee et al. 2019; Zhang et al. 2024a).

#### Applications of other microalgae in hypoxic tissues

Beyond *Chlorella*, other microalgae also show considerable promise in alleviating tumor and tissue hypoxia. It has also been reported that *Spirulina* can be used as a drug carrier instead of *Chlorella* (Liang et al. 2024a). A *Spirulina*-based drug delivery system (SpiD) has been developed for the treatment of osteosarcoma by combining chemotherapy and enhanced photodynamic therapy (An et al. 2024). *Synechococcus elongatus*, a unicellular cyanobacterium, is capable of photosynthetic O_2_ production across a wide range of wavelengths. *S. elongatus* has been utilized in ischemic hearts to improve cardiac function, where light, rather than blood, serves as fuel for cardiomyocytes (Cohen et al. 2017). These findings suggest that this direct application of cyanobacteria can enhance tissue oxygenation, protect myocardial metabolism, and increase cardiac output. To further boost radiotherapy efficiency, Chai et al. (2022) integrated two-dimensional ultrathin bismuth nanosheets as radiosensitizers onto *S. elongatus*, creating a biomimetic radiosensitization platform suitable for radioresistant hypoxic tumor environments (Chai et al. 2022). These studies encourage exploration of these photosynthetic microalgae for treating other hypoxic tissues. For instance, in cases of cerebral infarction due to impaired blood circulation, there exists a critical window for intervention during which local brain tissue suffers from ischemia and hypoxia. Providing sufficient nutrients to brain tissue within a few hours could enable recovery from cerebral infarction. This would mark a groundbreaking advancement in the use of microalgae-based systems for photosynthetic O_2_ production.

### Promoting postoperative wound healing

The healing of postoperative wounds is a prolonged process that is susceptible to infections, leading to amputation and pose life-threatening risks (de Andrade et al. 2022). Hypoxia is a key factor among the various elements that hinder the healing process. To solve this problem, several O_2_-releasing biomaterials have been clinically utilized, including hemoglobin, perfluorocarbons, and catalase (Han et al. 2023; Lee et al., 2022; Wang et al. 2017; Wang et al. 2024a). These materials have facilitated the development of various wound healing platforms, such as granules, hydrogels, and patch systems. Microalgae are natural photosynthetic microorganisms that produce O_2_ and can be integrated into wound dressings (Corrales-Orovio et al. 2023) (Fig. 1E). Additionally, they have the capability to synthesize and accumulate a range of high-value bioactive compounds, which facilitate the healing process by reducing inflammation, minimizing tissue damage, and promoting the expression of growth factors (Miguel et al. 2021). Among the wound healing treatments available, chronic wound recovery in diabetic patients presents a more significant challenge. This section highlights recent advancements in the use of live microalgae for treating diabetic wounds. We also discuss various methods for modifying microalgae, emphasizing their potential as promising candidates for future wound healing applications.

#### Harnessing microalgae for diabetic chronic wound healing

Choi et al. (2023) reported a biohybrid micro-robot system based on *C. reinhardtii*, which is coated with chitosan-heparin nanocomposites. This innovative system non-invasively penetrates thrombi to efficiently deliver O_2_ and eliminate chemokines, thereby accelerating the healing process of diabetic chronic wounds (Choi et al. 2023). Kang et al. (2024) developed a straightforward hydrogel by encapsulating active HEA algal strains within conventional GelMA gel, establishing a system for localized programmed treatment (Kang et al. 2024). This system can be programmed for multiple functions using light intensity modulation. It can emit high-intensity light for wound disinfection, and also provide low-intensity light for O_2_ production to alleviate hypoxia (Kang et al. 2024). Additionally, continuous light exposure can induce astaxanthin accumulation to remove excess ROS (Kang et al. 2024). Recently, a multifunctional covalent organic framework (COF) microalgal gel was created by harnessing the unique properties of hydrogels to treat chronic diabetic wounds (Jin et al. 2024). Notably, the COF binds with fibroblast growth factor to promote angiogenesis and reduce inflammatory responses while also interacting with microalgae to eliminate ROS and release dissolved O_2_, thereby alleviating wound hypoxia (Jin et al. 2024). Notably, some microalgae, such as *Chlorella*, can produce biohydrogen using solar energy under anaerobic conditions (Chen et al. 2022). The hydrogen released by these microalgae has the potential to neutralize harmful free radicals, serving as a protective mechanism against oxidative stress and inflammation (Chen et al. 2022). This offers an alternative strategy for developing novel materials for wound healing.

#### Alternative approaches of microalgae-base wound healing

The inability to effectively upregulate vascular endothelial growth factor (VEGF) is a significant factor that hinders angiogenesis and wound healing. To address this problem, Xing et al. (2025) combined the microalga *C. reinhardtii* with the antibacterial agent ciprofloxacin in a hydrogel. This created a composite gel that upregulates VEGF levels and promotes wound healing (Xing et al. 2025). Directly introducing VEGF is another viable strategy. Jia et al. (2024) successfully developed microalgal-loaded alginate hydrogel microspheres using microfluidic electrospray technology, which included VEGF to further enhance the therapeutic effects of wound treatment (Jia et al. 2024). Furthermore, genetically engineering microalgae to secrete VEGF or other crucial molecules that aid in wound regeneration can enhance their healing properties (Chavez et al. 2021, 2016; Corrales-Orovio et al. 2023). These approaches could pave the way for advanced wound healing therapies in the future.

Recently, 3D bioprinting has garnered increased attention for its applications in tissue repair and regeneration. Microalgae-based 3D bioprinting can also mitigate the limitations of hypoxia in printed materials. Wang et al. (2022) integrated O_2_-producing microalgae into the 3D printing process, resulting in scaffolds capable of generating sustainable O_2_ under light exposure. These scaffolds can adapt to irregularly shaped wounds and enhance the healing process (Wang et al. 2022). However, for larger wounds, surgical suturing remains the most direct and effective approach. Centeno-Cerdas et al. (2018) explored the inoculation of commercially available sutures with genetically engineered microalgae that can release specific recombinant bioactive molecules. They found that this photosynthetic gene therapy has the potential to produce next-generation bioactive sutures with enhanced healing capabilities (Centeno-Cerdas et al. 2018). It is important to note that the O_2_ production process by microalgae has dual effects. Moderate levels of O_2_ can promote wound healing, whereas excessive exposure may worsen inflammatory responses and damage tissue cells. Additionally, photosynthetic O_2_ production requires adequate light exposure. This necessitates precise control of light intensity or the use of specialized luminescent dressings. These factors are crucial for the future development of microalgae for wound healing.

### Immunotherapy applications of microalgae

Microalgae are considered to be organisms that do not trigger significant immune responses. This is mainly due to the fact that they lack key pathogen-associated molecular patterns recognized by the innate immune system, such as lipopolysaccharides and single-stranded RNA (Ma et al. 2024). This suggests that the human immune system may not have evolved the capacity to identify these cells as foreign entities that could trigger adverse rejection reactions (Ma et al. 2024). However, recent studies have revealed that microcystins found in microalga *Microcystis aeruginosa* share structural similarities with lipopolysaccharide endotoxins, which can function as immunostimulants (Meng et al. 2024). Microcystins may activate the cGAS-STING and IRF signaling pathways in dendritic cells, inducing the production of immune-stimulatory factors that promote tumor cell death through enhanced infiltration of cytotoxic CD8^+^ T cells. Notably, a variety of recombinant vaccine prototypes targeting both human and animal diseases have been developed using microalgae, yielding promising results in terms of production efficiency and immunoprotective effects (Marquez-Escobar et al. 2018; Ramos-Vega et al. 2021). Additionally, leveraging the specific binding capabilities between antigens and antibodies within immune responses can enhance the drug delivery potential of microalgae. For example, Delalat et al. (2015) reported that the engineered the diatom *T. pseudonana* can bind to cell-targeting antibodies through expressing the IgG-binding domain of protein G on its biogenic silica surface (Delalat et al. 2015). This innovation facilitates the delivery of chemotherapy drugs encapsulated within nanoporous diatoms directly to cancer cells, representing a novel strategy with significant potential for advancing microalgal immunotherapy.

## Challenges and prospects

### Development of smart microalgae systems for targeted therapies

Although it has been demonstrated that microalgae are potential for targeted drug delivery (Li et al. 2024b; Akolpoglu et al. 2020), current control strategies primarily rely on magnetically guided Spirulina or photo-targeted *C. reinhardtii*. These approaches inevitably risk mechanical or thermal damage to surrounding healthy tissues, particularly in deep-seated tumor therapy. Future microalgal engineering should consider to integrate materials science and synthetic biology to develop advanced therapeutic systems. For instance, programming microalgae to activate in response to tumor-specific signals (e.g., pH changes, enzymes), reducing dependence on external devices. Alternatively, genetic modifications such as photosensitive protein expression could enhance near-infrared targeting—eliminating invasive light-access surgeries (e.g., thoracotomy) and fiber-optic delivery systems, thereby minimizing surgical trauma, complications, and costs.

### Evaluating long-term biosafety to advance clinical translation

It has been demonstrated that microalgae can be metabolized and cleared through hepatic and renal pathways in vivo. However, certain components may still pose biosafety concerns that require careful evaluation. For instance, microcystins, toxins typically produced by cyanobacteria, demonstrate potent hepatotoxicity and carcinogenicity, limiting the clinical use of certain microalgae. While toxicity risks could be mitigated through dose optimization and tumor-localized targeting, most current studies remain confined to animal models, necessitating further validation of metabolic pathways and long-term accumulation effects. Future efforts could employ genetic engineering to eliminate cyanotoxins, enhancing clinical applicability. Additionally, microalgae-nanomaterial hybrids (e.g., Fe_3_O_4_-coated magnetic systems) can improve drug-targeting efficiency but raise toxicity concerns due to organ accumulation. A rigorous benefit-risk assessment is thus imperative before clinical adoption.

### Optimizing microalgal robot fabrication for scalable production and application

Although microalgae can be loaded with drugs via electrostatic adsorption, limitations in efficiency due to surface charge variability and cell wall structure will cause payload dispersion during delivery (Zhong et al. 2021b). Covalent conjugation may address these issues by enhancing drug loading capacity and stability, however, the cost and complexity of fabrication will be increased (Zhang et al. 2022c). Therefore, integrating complementary strategies in microalgae is critical to advance microalgal robot production. This necessitates developments in the following areas: (i) leveraging autofluorescence for real-time tracking and targeting optimization; (ii) applying biodegradable nanocoatings to reduce toxicity and processing costs; (iii) integrating chemo-, radio-, or photothermal therapies for synergistic effects; and (4) fostering industrial partnerships to enable cost-effective, scalable production.

## Conclusion

Here we summarized the applications of microalgae as natural multifunctional biomaterials in the field of biomedicine. This review highlights the latest research advancements in areas such as medicinal health, drug delivery, hypoxia-related tumors, wound healing, and immunotherapy. Microalgae are abundant in bioactive components and possess unique directional properties along with easily modifiable surface structures. They exhibited excellent biocompatibility, accessibility, and low cultivation costs, positioning them as promising emerging biomaterials. However, further studies are needed to understand how biohybrid robots made from microalgae can effectively navigate and precisely target the complex and variable environments within the human body. Moreover, it is important to explore more unconventional microalgae strains or engineered strains that useful for biomedical application. These complex and extensive undertaking will require collaboration across various research fields, including biology, clinical medicine, materials science, chemistry and physics. Moreover, additional clinical evaluations are necessary to assess potential toxicity in metabolic pathways within the body. Overall, microalgae have shown remarkable multifunctionality in biomedicine, and they have the potential to revolutionize drug delivery and targeted therapies in biopharmaceuticals in the near future.

## Data Availability

The data that support the findings of this study are available from the corresponding author upon reasonable request.
